# Towards an Integrated Healthcare System: Evolutionary Game Analysis on Competition and Cooperation Between Urban and Rural Medical Institutions in China

**DOI:** 10.3389/fpubh.2022.825328

**Published:** 2022-03-10

**Authors:** Xinglong Xu, Jiajie Liu, Sabina Ampon-Wireko, Henry Asante Antwi, Lulin Zhou

**Affiliations:** ^1^School of Management, Jiangsu University, Zhenjiang, China; ^2^Medical Insurance and Public Policy Research Center, Jiangsu University, Zhenjiang, China

**Keywords:** competition and cooperation, evolutionary game, strategy, rural medical institutions, urban medical institutions

## Abstract

**Background:**

The game of interest is the root cause of the non-cooperative competition between urban and rural medical and health institutions. The study investigates competition and cooperation among urban and rural medical institutions using the evolutionary game analysis.

**Methods:**

With the evolutionary game model, analysis of the stable evolutionary strategies between the urban and rural medical and health facilities is carried out. A numerical simulation is performed to demonstrate the influence of various values.

**Results:**

The result shows that the cooperation mechanism between urban and rural medical Institutions is relevant to the efficiency of rural medical institutions, government supervision, reward, and punishment mechanism.

**Conclusions:**

Suggestions for utilizing the government's macro regulation and control capabilities, resolving conflicts of interest between urban and rural medical and health institutions is recommended. In addition, the study again advocates mobilizing the internal power of medical institutions' cooperation to promote collaboration between urban and rural medical and health institutions.

## Background

China's medical institutions are divided into three levels (1). The first level hospital refers to the medical and health institutions located in urban communities or villages, which are mainly responsible for providing prevention, medical treatment, health care, rehabilitation and other services to community residents. Secondary hospitals usually refer to regional hospitals, which are mainly responsible for the treatment and referral services of general diseases of residents within their jurisdiction. Tertiary hospitals usually refer to provincial and municipal hospitals, which are mainly responsible for the treatment, teaching and research of difficult and miscellaneous diseases. For a long time, the Chinese government has been committed to building a reasonable and orderly hierarchical diagnosis and treatment system, but it lacks mandatory constraints on Residents' medical behavior. Therefore, with the economic and social development and the improvement of residents' health needs, Chinese residents are more inclined to directly choose urban hospitals and ignore rural medical and health institutions, which leads to a waste of resources and aggravates the economic burden and accessibility of residents' medical treatment. To carefully allocate medical and health resources and fair access to essential medical and health services, the State Council on Deepening the Reform of the Medical and Health System (SCRMHS) in 2009 pointed out that medical and health reform must build a structure for the division of labor and cooperation between urban and rural medical and health institutions. This would ensure equitable access to essential medical and health services while strategically allocating medical and health resources. In recent years, local government departments have increased economic investment in primary medical and health institutions and explored the establishment of cooperation models including medical unions and medical groups to promote the development and capacity improvement of primary medical and health institutions. However, the actual operation effect is not ideal. The fragmentation of the medical service system, disorder of patients' medical treatment, excessive waste of medical resources, and other problems are still prominent. In 2015, the general office of the state council issued the guiding opinions on promoting the construction of hierarchical diagnosis and treatment system. It further indicated that it is necessary to fully play the role of different host medical and health institutions in constructing the division of labor and cooperation mechanism between urban and rural medical and health institutions. In government spending, however, the overall shortage improves residents' health needs, whose medical and health institutions at different levels such as background, the majority of urban and rural medical and health institutions, not collaborative competition. Along the same line, each professional autonomy is the main body in the system to find their development to the induced demand, excessive or selective providing medical services. This kind of disorderly competition directly leads to the waste of medical resources and decreased system efficiency. Thus, theoretically, understanding scientific analytic co-opetition relationships between urban and rural medical and health institutions will help promote urban and rural medical and health institutions. Additionally, it will assist in alleviating the challenges faced by the public in receiving healthcare and increasing the efficiency with which medical and health resources.

Following a literature survey, foreign scholars are exploring and practicing the division of labor and cooperation mechanism between urban and rural medical and health institutions. Niskanen (2) and Jeroen et al. (3) applied the integration theory to the medical service system because of the hierarchical fracture phenomenon between urban and rural medical and health institutions. We will explore the establishment of a continuous and collaborative mechanism among medical and health institutions. Enthoven and Laura (4) established that different levels of health care institutions need to integrate personnel, equipment, information, management, and services to provide patients with efficient, safe, and interconnected integrated health services. The British scholar Fulop et al. (5) classified the elements of collaboration among medical and health institutions into five categories: functional, organizational, professional, clinical, and normative. But how to collaborate, Hasting et al. (6) believed that collaboration factors were important carriers of collaboration between urban and rural medical and health institutions. The cooperation efficiency of elements should be improved from the perspective of combining government and market. Erica et al. (7) also analyzes the process and tools of collaboration and emphasizes the vital role of personnel in cooperation. With the rapid development of Internet electronic information platform construction and information technology, Svensson (8) proposed that collaboration between professionals from different healthcare organizations could be supported through Information technology systems. Association between medical and health institutions. In the process of healthcare governance and partnership, Lim and Lin (9), a Scholar from Hong Kong, China, pointed out that the government should play a key role in coordinating the concerns and priorities of relevant parties to achieve health goals. In terms of specific collaboration content, Lemetti et al. (10) revealed the importance of collaboration between the hospital and primary health care providers for continuity of care for elderly patients. Sheehan et al. (11) found that communication and coordination between hospitals and primary care institutions can ensure comprehensive people-oriented care. Otte et al. (12) points out that available evidence shows that effective collaboration between general practitioners and hospital doctors can lead to measurable health benefits, better use of resources, improved patient experience, and improved health outcomes.

Chinese scholars' research on the division and cooperation of urban and rural medical and health institutions began after the “new medical reform” policy promulgated in 2009. Wang and Wanwen (13) points out that the urban hospitals and community health service institutions of all kinds of medical and health resources have the quantity and quality of the imbalances to varying degrees. Hospital health resources utilization efficiency is lower than the community health service institutions, It reveals the necessity of the division of labor and cooperation between urban and rural medical institutions. Gao (14) affirmed the positive role of collaboration between urban and rural medical and health institutions, holding that it is not only conducive to ensuring the quality of medical services but also improve the utilization efficiency of medical resources, and is the leading way to ensure the sustainable development of medical service system. In terms of cooperation mode, Lu and Qian (15) summarized the cooperation mode into three types, i.e., loose, semi-tight, and tight, according to the cooperation practices among medical and health institutions in some regions of China, such as “medical community,” “regional medical consortium”, medical group and regional health cooperation network. Liu and Qunzhao (16) noted that various regions have investigated the establishment of a division of labor and cooperation mechanism between urban and rural medical and health institutions to implement corresponding medical reforms. The results have been insignificant due to a lack of relevant policy guarantees, oversight and assessment of division of labor and cooperation, and an imperfect incentivization system. To explore a more effective Collaborative path, Yuan et al. (17) proved that the medical alliance mode with trustee-taking as the core integration measure has closer cooperation in the dimension of governance structure, especially in the three indicators of leadership style, innovation, support, and connectivity. Zhou et al. (18) asserted that the capitation payment method could lead medical institutions to generate the internal motivation of up-down linkage, conducive to promoting the integration of medical and health institutions. Zheng (19) proposed that top-level design should be emphasized to integrate existing medical service systems to promote collaboration between urban and rural medical and health institutions.

In conclusion, scholars have conducted extensive discussion and analysis of medical and health institutions at various levels, primarily due to the necessity of urban and rural medical and health institutions, elements, patterns, and paths. Nonetheless, there is little research on the competitive relationship between the in-depth analysis, the internal mechanism of urban and rural medical and health institutions. The classical economic theory believes that the essence of the social division of labor is to obtain more economic profits, and it is the balance of cooperation and game among stakeholders (20). Evolutionary game theory overcomes the problem that completely rational game analysis is divorced from reality and is more practical. After the concept of “Evolutionary Stable Strategy” was introduced by Maynard and Price (21), the application of Evolutionary game theory developed rapidly. Widely used in ecology, economics, social physics and so on. For example, Guttman (22) applied evolutionary game theory to study whether reciprocity can exist in groups with opportunism. Yuhong and Zhong (23) used two evolutionary game models to describe the competition and cooperation between intermediary enterprises. In terms of the applicability of research objects, Xiang et al. (24) pointed out that urban and rural medical and health institutions are a service system integrating medical treatment, public health, drug supply, and rehabilitation nursing, with dual attributes of general welfare and competitiveness. However, under the background of the infinite expansion of urban hospitals and the slow development of community health service institutions, the goals of medical and health institutions are not consistent, and it is difficult for the autonomous subjects of various specialties in the system to achieve practical cooperation to obtain their profits. The dilemma is at the heart of the inability of urban and rural medical and health organizations to cooperate (25). Additionally, because public medical institutions serve as the primary body for general social welfare, government policy changes tend to impact the strategic choices made by medical institutions directly. As a result, medical and health institutions at various levels will choose their respective strategies to meet patient needs. Therefore, it can be seen that the competition and cooperation relationship between urban and rural medical and health institutions has prominent evolutionary game characteristics.

Thus, what interest game occurs between urban and rural medical and health institutions, and what critical influencing factors exist in this process? Based on this, this article will use the primary method of evolutionary game theory to play a game between urban and rural medical and health institutions. It will also employ evolutionary game theory to investigate an innovative collaboration between medical and health institution's problems. Moreover, the study will consider government as an environmental element in the consideration process but will not consider its policy implications for medical and health institutions or constraints. This shift benefits actors by enhancing their leading role in mechanism development.

## Methods

### Game Relationship Analysis

To protect social welfare and retain their operation and development, urban and rural medical and health institutions have the option of cooperating or not cooperating. The mechanism of collaboration between the two parties is mainly expressed in the ability of urban and rural health institutions to agree on the policies or pathways defined by the government. On the one hand, the implementation of government policies must depend on the cooperation of urban and rural medical and health institutions. The development of urban and rural health institutions relies on the government policy incentive and security mechanism. On the other hand, the government departments have the power and responsibility to control the medical and health institutions. In contrast, the medical and health institutions follow the social public welfare characteristics stipulated by the government departments. The non-cooperation between the two sides is mainly due to the differences in rational level and objectives.

Generally speaking, government departments consider the scarcity of available medical and health resources and regional disparities and will exercise macro-control over the long term, with the primary goal of ensuring universal access to essential health services. Additionally, they consider sustainable development for all; medical and health institutions and prioritize the development of hospital operations. Therefore, the two sides' cooperation is primarily reflected in the Macro and Micro levels. At the macro level, collaboration can effectively improve the operation efficiency of the medical service system, reduce service costs, satisfy residents' desire for rapid and convenient diagnosis and treatment, maximize resource allocation and utilization efficiency, and ensure that everyone has access to essential medical and health services.

At the micro-level, collaboration can effectively relieve urban hospitals of pressure, prevent conflicts between doctors and patients, and increase their effectiveness in researching and treating rare and major diseases; community health service institutions can also fully exploit their inherent geographical advantages, strengthening treatment of common diseases (26).

The competition between urban and rural medical and health institutions is also ubiquitous. In the realistic background, with their advantages in talent, technology, instruments, and equipment, urban hospitals attract many patients to go to the city hospital directly. Meanwhile, to pursue economic profits, urban hospitals cannot carry out the transformation in time during the recovery period of patients. To deal with the problems of “No.1 difficult to ask for” and “difficult to see a doctor,” the scale of urban hospitals is expanding infinitely, and medical. Health resources and patients flow further to urban hospitals, which improves the business income of urban hospitals to a certain extent, but also increases the investment cost and management difficulty of urban hospitals, At the same time, it has caused the problems of the insufficient business volume of community health service institutions and excessive waste of medical and health resources (27). Under the government's financial input and control, community health service institutions pay attention to health prevention and health promotion and take measures to improve the proportion of reimbursement for patients, which improves the competitiveness of grass-roots medical institutions.

However, their ability to provide medical services, economic revenue, medical environment, and development space is still behind metropolitan hospitals. Doctors' desire to visit community health service institutions is low, and the grass-roots diagnosis rate is not guaranteed.

Therefore, it is easy to adopt vicious competition among different institutions (publishing false advertisements and seizing the medical market) (28). In conclusion, under the background of market economy system reform, urban hospitals occupy an absolute advantage in medical service, which covers a large number of medical and health resources and patients.

This is not difficult to comprehend due to the lack of a joint benefit coordination and distribution mechanism between urban hospitals and community health service institutions. Moreover, each subject in the process possesses the characteristics of a rational economic person, and it is challenging to realize the sustainable development of the division of labor and cooperation. Nevertheless, public health institutions retain the characteristics of general social welfare while also bearing the burden of ensuring that everyone has access to essential medical and health services. As such, the government must exercise macro-control.

Thus, it can be deduced that the rational level of urban and rural medical and health institutions should be limited rationality. Their strategic responses will be influenced by changes in their environment and government policies, which tends to balance out over time, eventually evolving into a stable game strategy.

### Basic Assumptions

According to the functional orientation of China's three-level medical and health institutions, the Chinese government has been committed to guiding residents to visit rural medical and health institutions firstly, which can increase residents' access to medical treatment and reduce medical expenses, but there are no mandatory measures. Therefore, in the context of economic and social development and the improvement of residents' health needs, more residents tend to choose urban hospitals for treatment, regardless of the type of disease, which will increase the income level of hospitals. Therefore, as a rational economic man, urban hospitals have reason to accept more patients for treatment, but this will lead to expensive and difficult medical treatment, and waste medical and health resources. Therefore, the model mainly takes urban hospitals and rural medical and health institutions as two types of subjects, and constructs the corresponding model according to their behavior strategies.

The quality of rational economic man and social public welfare services is a springboard for cooperative tactics between urban and rural medical and health institutions. Therefore, developing a system for coordinating and allocating mutual interests is critical for promoting cooperation between urban and rural medical and health institutions that rely on government macro-control methods. As a result, the following hypotheses are proposed:

#### Hypothesis 1

In 2015, the Chinese government issued a policy to strengthen the division of labor and cooperation of medical institutions. In 2017, it proposed the construction requirements of “Medical Consortium”, which aims to integrate urban hospitals and rural medical and health institutions into the community of interests of medical groups. This can promote the cooperative relationship between urban hospitals and rural hospitals, but it is not mandatory. The strategy defined for collaboration between metropolitan hospitals and community health service institutions is supposed to be collaboration, not cooperation. The probability of “cooperation” strategy adopted by urban hospitals and community health service institutions is *x* and *y*, and the likelihood of “non-cooperation” is 1−*x* and 1−*y*; Of which 0 ≤ *x* ≤ 1, 0 ≤ *y* ≤ 1; the costs invested in the two normal states are *C*_1_ and *C*_2_, Income are *E*_1_ and *E*_2_, In addition, under normal conditions, the government strengthens financial subsidies to the grass-roots level, which is set as *G*_5_.

#### Hypothesis 2

Most hospitals in China are still run by the government and have the characteristics of public welfare, but they also retain the nature of operation in the process of hospital operation. Therefore, as rational economic people, urban hospitals and rural medical and health institutions tend to attract more patients to meet their own interest needs. It is assumed that both urban hospitals and community health service institutions maximize economic profits. The cost of vicious competition in urban hospitals and community health service institutions are *C*_3_ and *C*_4_, Income is *E*_3_ and *E*_4_; At this time, it is further assumed that the government will supervise the public welfare of medical and health institutions, and the probability of supervision is *a*(0 < *a* <1), The penalties for violations by urban hospitals and community health service institutions are as follows: *G*_1_ and *G*_2_.

#### Hypothesis 3

In recent years, the Chinese government has always emphasized the public welfare nature of hospitals, adhered to the fundamental purpose of focusing on people's health, and strengthened the division of labor and cooperation between urban hospitals and rural medical and health institutions. It is assumed that both urban hospitals and community health service institutions aim to undertake public social welfare. The costs of the two collaborative inputs are *C*_5_ and *C*_6_, Income is *E*_5_ and *E*_6_; At this time, the patient's medical efficiency is improved, the medical and health resources have been fully allocated, and the government's performance is improved. Therefore, the government will increase the compensation for urban hospitals and community health service institutions, which are set as *G*_3_ follows.

#### Hypothesis 4

Urban hospitals and rural medical and health institutions as two types of subjects with competitive relationship, their behavior strategy choice will affect the final result of urban and rural medical and health institutions, so their strategy choice is not necessarily the same. Suppose that one party chooses the non-cooperation strategy and the other party chooses the cooperation strategy. Through vicious competition, one party will excessively seize the source of disease. In contrast, the other party's income will be reduced accordingly (this impact can be ignored when both parties adopt the non-cooperation strategy). The reduced income is set as *E*_7_. According to the above assumptions and the dependence of the techniques of urban and rural medical and health institutions, the evolutionary model of cooperation between urban hospitals and community health service institutions established by using the game benefit matrix is shown in Table 1.

**Table 1:** Payment matrix of cooperation strategy selection game between urban hospitals and community health service institutions.

		**Community health service institutions**	
		**Cooperation**	**No collaboration**
**City hospital**	**Cooperation**	*E*_1_+*E*_5_+*G*_3_−*C*_1_−*C*_5_	*E*_1_−*E*_7_−*C*_1_
		*E*_2_+*E*_6_+*G*_4_+*G*_5_−*C*_2_−*C*_6_	*E*_2_+*E*_4_+*G*_5_−*C*_2_−*C*_4_−*aG*_2_
	**No collaboration**	*E*_1_+*E*_3_−*C*_1_−*C*_3_−*aG*_1_	*E*_1_+*E*_3_−*C*_1_−*C*_3_−*aG*_1_
		*E*_2_+*G*_5_−*E*_7_−*C*_2_	*E*_2_+*E*_4_+*G*_5_−*C*_2_−*C*_4_−*aG*_2_

### Construction of Model

In the game model of urban hospitals and community health service institutions, the expected benefits of urban hospitals choosing “cooperation” and “non-cooperation” strategies are *U*_11_ and *U*_12_, the average expected return is then there are:


$$\begin{array}{l} U_{11}=y(E_{1}+E_{5}+G_{3}-C_{1}-C_{5})+(1-y)(E_{1}-E_{7}-C_{1}),\\ U_{12}=y(E_{1}+E_{3}-C_{1}-C_{3}-aG_{1})+(1-y)(E_{1}+E_{3}-C_{1}\\ \;-C_{3}-aG_{1}),\\ U_{1}=xU_{11}+(1-x)U_{12}. \end{array}$$

Similarly, the expected benefits of community health service institutions choosing “cooperation” and “non-cooperation” strategies are *U*_21_ and *U*_22_, The average expected return is *U*_2_, then there are:


$$\begin{array}{l} U_{21}=x\left(E_{2}+E_{6}+G_{4}+G_{5}-C_{2}-C_{6}\right)\\ \;+\left(1-x\right)\left(E_{2}+G_{5}-E_{7}-C_{2}\right),\\ U_{22}=x\left(E_{2}+E_{4}+G_{5}-C_{2}-C_{4}-aG_{2}\right)\\ \;+\left(1-x\right)\left(E_{2}+E_{4}+G_{5}-C_{2}-C_{4}-aG_{2}\right),\\ U_{2}=yU_{21}+\left(1-y\right)U_{22}. \end{array}$$

The dynamic replication equation of urban hospitals and community health service institutions is:


$$\left\{ \begin{array}{l} F(x)=\frac{dx}{dt}=x(U_{11}-U_{1})=x(1-x)[y(E_{5}+G_{3}-C_{5}+E_{7})\\ \;-E_{7}-E_{3}+C_{3}+aG_{1}],\\ F(y)=\frac{dy}{dt}=y(U_{21}-U_{2})=y(1-y)[x(E_{6}+G_{4}-C_{6}+E_{7})\\ \;-E_{7}-E_{4}+C_{4}+aG_{2}]. \end{array}\right.$$

Therefore, the Jacobian matrix of equation (1) is:


$$\left[\begin{array}{l} \left(1-2x)[y(E_{5}+G_{3}-C_{5}+E_{7})(x-x^{2})(E_{5}+G_{3}-C_{5}+E_{7}\right)\\ \;-E_{7}-E_{3}+C_{3}+aG_{1}]\;\\ (y-y^{2})(E_{6}+G_{4}-C_{6}+E_{7})\;(1-2y)[x(E_{6}+G_{4}-C_{6}\\ \;+E_{7})-E7-E_{4}+C_{4}+aG_{2}] \end{array}\right]$$

To make the benefits of both sides of the game closer to reality, relevant constraints need to be added. When urban hospitals adopt the “cooperation” strategy, the business volume of community health service institutions is increased, and the service capacity is also improved accordingly. Therefore, community health service institutions are motivated to adopt the “cooperation” strategy. Consequently, it is stipulated that the income of community health service institutions when assuming the “cooperation” strategy is more significant than that when adopting the “non-cooperation” strategy, then (*E*_2_+*E*_6_+*G*_4_+*G*_5_−*C*_2_−*C*_6_)>(*E*_2_+*E*_4_+*G*_5_−*C*_2_−*C*_4_−*aG*_2_).

When urban hospitals adopt the “non-cooperation” strategy, the benefits of community health service institutions adopting the “non-cooperation” strategy for their development are also realistic. At this time, the benefits of “non-cooperation” of community health service institutions are greater than those under “cooperation”, then there is (*E*_2_+*E*_4_+*G*_5_−*C*_2_−*C*_4_−*aG*_2_)>(*E*_2_+*G*_5_−*E*_7_−*C*_2_).

Similarly, it is reasonable that when community health service institutions adopt the “cooperation” strategy, the benefit of urban hospitals adopting the “cooperation” strategy is greater than that of “non-cooperation”. There is (*E*_1_+*E*_5_+*G*_3_−*C*_1_−*C*_5_)>(*E*_1_+*E*_3_−*C*_1_−*C*_3_−*aG*_1_), when the community health service institutions adopt the “non-cooperation” strategy, the benefit of the “non-cooperation” strategy is greater than that of the “cooperation”, which is in line with the reality, then there is (*E*_1_+*E*_3_−*C*_1_−*C*_3_−*aG*_1_)>(*E*_1_−*E*_7_−*C*_1_).

Through the above analysis, it can be further calculated that: (*E*_2_+*E*_6_+*G*_4_+*G*_5_−*C*_2_−*C*_6_)>(*E*_2_+*G*_5_−*E*_7_−*C*_2_), (*E*_1_+*E*_5_+*G*_3_−*C*_1_−*C*_5_)>(*E*_1_−*E*_7_−*C*_1_).

According to the copied dynamic equation, in the plane *M* = {(*x, y*)|0 ≤ *x* ≤ 1, 0 ≤ *y* ≤ 1}, it is concluded that there are five local equilibrium points in the system composed of urban hospitals and community institutions: *D*_1_(0, 0), *D*_2_(0, 1), *D*_3_(1, 0), *D*_4_(1, 1), $D_{5}\left(\frac{E_{7}+E_{4}-C_{4}-aG_{2}}{E_{6}+G_{4}-C_{6}+E_{7}},\frac{E_{7}+E_{3}-C_{3}-aG_{1}}{E_{5}+G_{3}-C_{5}+E_{7}}\right)$. The equilibrium point is verified by the local stability analysis method of the Jacobian matrix, and the results are shown in Table 2.


$$\begin{array}{l} T=-\left[\frac{E_{7}+E_{4}-C_{4}-aG_{2}}{E_{6}+G_{4}-C_{6}+E_{7}}-{\left(\frac{E_{7}+E_{4}-C_{4}-aG_{2}}{E_{6}+G_{4}-C_{6}+E_{7}}\right)}^{2}\right]\\ \;\left(E_{5}+G_{3}-C_{5}+E_{7}\right)[\frac{E_{7}+E_{3}-C_{3}-aG_{1}}{E_{5}+G_{3}-C_{5}+E_{7}}\\ -{\left(\frac{E_{7}+E_{3}-C_{3}-aG_{1}}{E_{5}+G_{3}-C_{5}+E_{7}}\right)}^{2}](E6+G4-C6+E7) \end{array}$$

**Figure 1:**
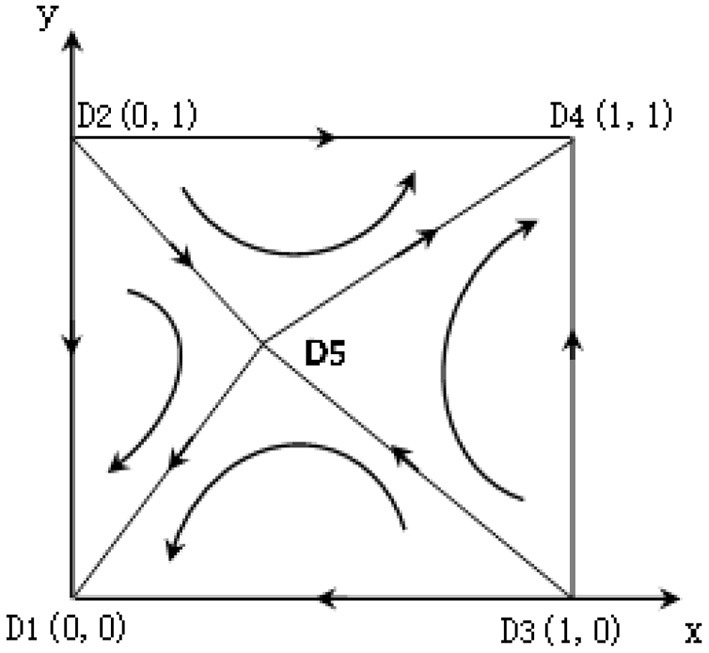
Dynamic process of the game between urban hospitals and community health service institutions.

It can be seen from Table 2 that two of the five equilibrium points in the system are stable; that is, urban hospitals and grass-roots hospitals choose cooperation; Urban hospitals and community health service institutions all choose the strategy of non-cooperation; in addition, *D*_2_(0, 0), *D*_3_(1, 0) are the unbalance points. $D_{5}\left(\frac{E_{7}+E_{4}-C_{4}-aG_{2}}{E_{6}+G_{4}-C_{6}+E_{7}},\frac{E_{7}+E_{3}-C_{3}-aG_{1}}{E_{5}+G_{3}-C_{5}+E_{7}}\right)$ is saddle point (as shown in Figure 1), The connected broken lines by *D*_2_, *D*_3_, *D*_5_ can be regarded as the critical value of the system converging to different modes: Above the polyline is the probability of combining to the ideal method *D*_4_(1,1); Below the broken line is the probability of connecting to the wrong way *D*_1_(0,0), According to the expression of the saddle point, the change of relevant parameters will cause the movement of the saddle point, to regulate the evolution direction.

**Table 2:** Local stability analysis results.

**Equilibrium point**	**DetJ**		**Tr**		**result**
D_1_	(−*E*_7_−*E*_3_+*C*_3_+*aG*_1_) (−*E*_7_−*E*_4_+*C*_4_+*aG*_2_)	+	(−*E*_7_−*E*_3_+*C*_3_+*aG*_1_)+ (−*E*_7_−*E*_4_+*C*_4_+*aG*_2_)	-	ESS
D_2_	(*E*_5_+*G*_3_−*C*_5_−*E*_3_+*C*_3_+*aG*_1_) (*E*_7_+*E*_4_−*C*_4_−*aG*_2_)	+	(*E*_5_+*G*_3_−*C*_5_−*E*_3_+*C*_3_+*aG*_1_)+ (*E*_7_+*E*_4_−*C*_4_−*aG*_2_)	+	Unstable point
D_3_	(*E*_7_+*E*_3_−*C*_3_−*aG*_1_) (*E*_6_+*G*_4_−*C*_6_−*E*_4_+*C*_4_+*aG*_2_)	+	(*E*_7_+*E*_3_−*C*_3_−*aG*_1_)+ (*E*_6_+*G*_4_−*C*_6_−*E*_4_+*C*_4_+*aG*_2_)	+	Unstable point
D_4_	(−*E*_5_−*G*_3_+*C*_5_+*E*_3_−*C*_3_−*aG*_1_)+ (−*E*_6_−*G*_4_+*C*_6_+*E*_4_+*C*_4_+*aG*_2_)	+	(−*E*_5_−*G*_3_+*C*_5_+*E*_3_−*C*_3_−*aG*_1_)+ (−*E*_6_−*G*_4_+*C*_6_+*E*_4_+*C*_4_+*aG*_2_)	-	ESS
D_5_	*T*	-	0		Saddle point

### Parameter Analysis

In the following, the main parameters affecting the system evolution in the game between urban hospitals and community institutions will be analyzed according to the expression of the saddle point, and the linkage response of parameters will not be considered here.

1. Parameters *C*_3_, *C*_4_, *C*_5_, *C*_6_. The area above the broken line becomes more prominent when the cost *C*_3_ of urban hospitals to adopt the means of vicious competition increases, moves vertically downward, or when the cost *C*_4_ of community health service institutions to adopt the standards of fierce competition increases, *D*_5_ moves horizontally to the left shows that the rise in input cost is conducive to the benign evolution of the system when urban hospitals or community health service institutions adopt vicious competition; When the input cost *C*_5_ of urban hospitals increases after the cooperation of urban and rural medical and health institutions, *D*_5_ moves vertically, or when the input cost *C*_6_ of community health service institutions increases after the collaboration of urban and rural medical and health institutions, moves horizontally to the right, which makes the area above the broken line smaller, indicating that increasing the input cost of cooperation of urban and rural medical institutions is not conducive to the benign evolution of the system.
2. Parameters *G*_1_, *G*_2_. When the punishment *G*_1_ of urban hospitals taking means of vicious competition under government supervision increases, *D*_5_ moves vertically downward. When the sentence *G*_2_ of community health service institutions taking means of ferocious competition under government supervision increases, *D*_5_ moves horizontally to the left. The area above the broken line becomes more prominent, it shows that the government increases the punishment of vicious competition for urban hospitals or community health service institutions, which is conducive to the benign evolution of the system.
3. Parameters *E*_3_, *E*_4_, *E*_5_, *E*_6_, *E*_7_. When the income of *E*_3_ urban hospitals adopting the means of vicious competition increases, *D*_5_ moves vertically, or when the payment of community health service institutions after adopting the standards of fierce competition *E*_4_ increases, *D*_5_ moves horizontally to the right, both reduce the area above the broken line and reduce the probability of system evolution to *ESS* stable point *D*_4_, indicates an increase of income from vicious competition in urban hospitals or community health service institutions is not conducive to the benign development of the system; When the payment *E*_5_ of urban hospitals increases after the cooperation of urban and rural medical and health institutions, *D*_5_ moves vertically downward, or when the income *E*_6_ of community health service institutions increases after the collaboration of urban and rural medical and health institutions, *D*_5_ moves horizontally to the left, both increase the area above the broken line and increase the probability of system evolution to *ESS* stable point *D*_4_, demonstrates an increase of income after the cooperation between urban hospitals and community health service institutions is conducive to the benign development of the system; When one party of the urban hospital or community health service institution adopts the means of vicious competition to increase the income *E*_7_ reduced by the other party, *D*_5_ moves upward to the right, making the area above the broken line smaller, indicating that the more the gain reduced by the other party by either party of the urban hospital or community health service institution adopts the means of vicious competition, the more it is not conducive to the benign evolution of the system.

## Results

### Numerical Experiment and Result Analysis

According to the constraint conditions and replication dynamic equation, the numerical experiment is carried out on the evolution process of the interaction behavior between urban hospitals and community health service institutions by using MATLAB tools to analyze the impact of the changes of parameters such as the initial proportion of a particular strategy selected by the system (*x, y*), the government supervision probability (*a*), the government subsidy (*G*_3_, *G*_4_) after the cooperation between urban hospitals and community health service institutions on the evolution results.

**Figure 2:**
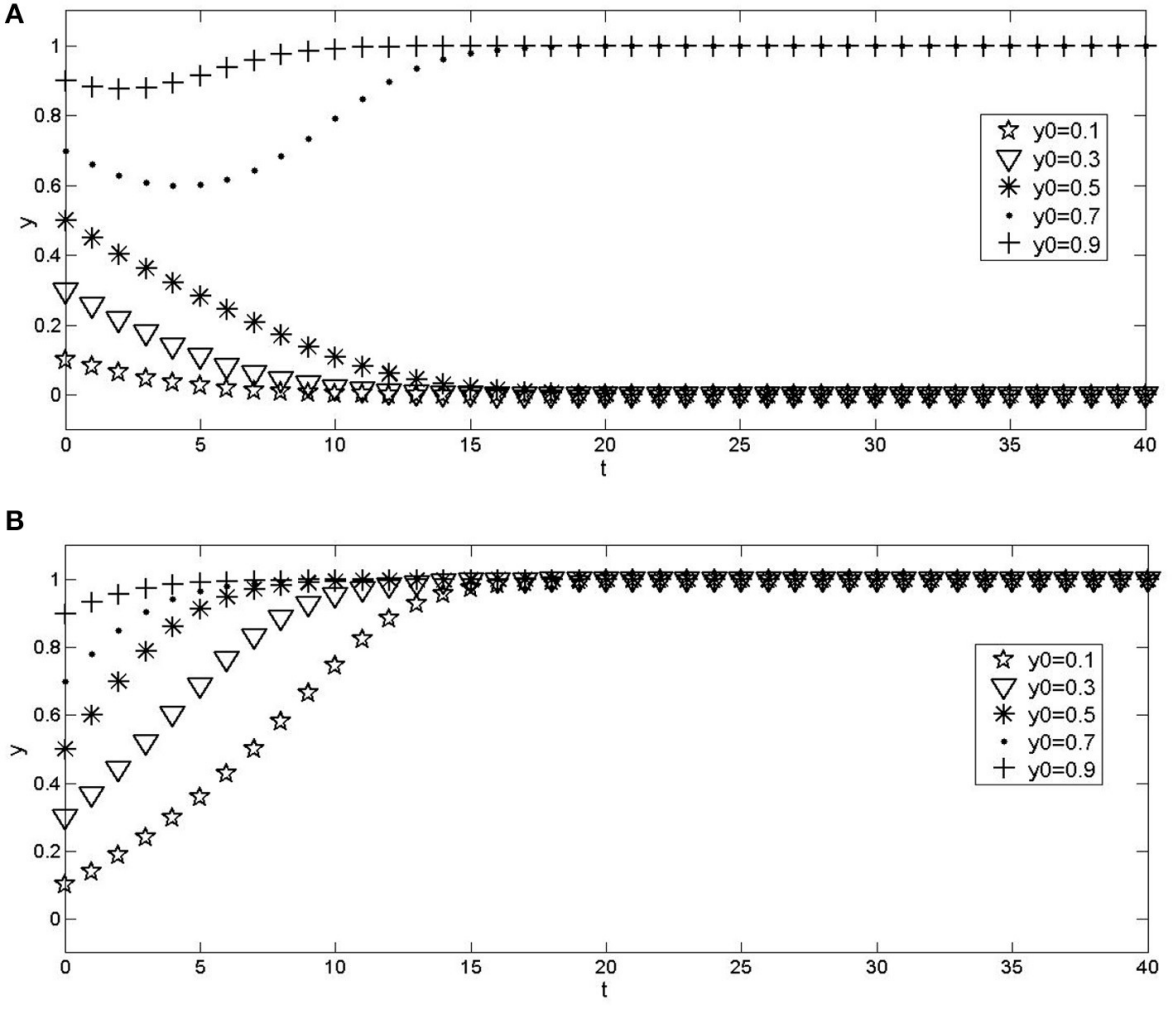
Effects of different initial proportions of institutions that choose a strategy on evolution results. **(A)** x = 0.2; **(B)** x = 0.8.

**Figure 3:**
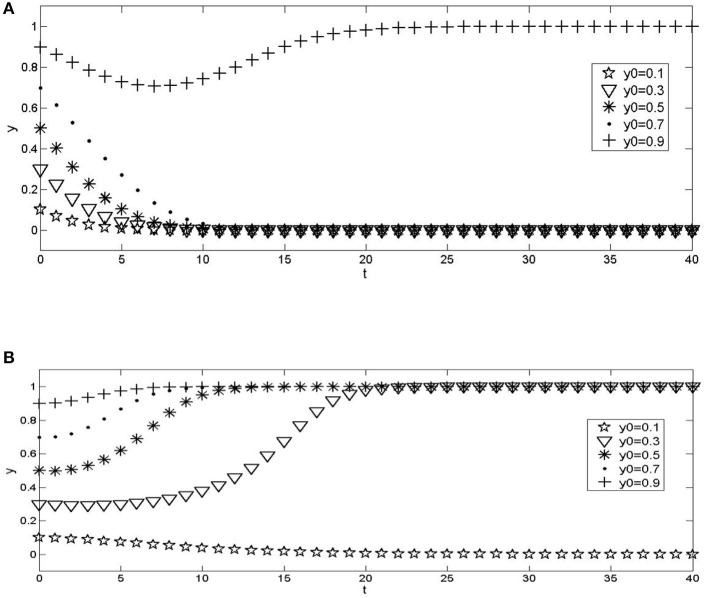
Impact of changes in government supervision probability on evolution results. **(A)** a = 0.2; **(B)** a = 0.8.

**Figure 4:**
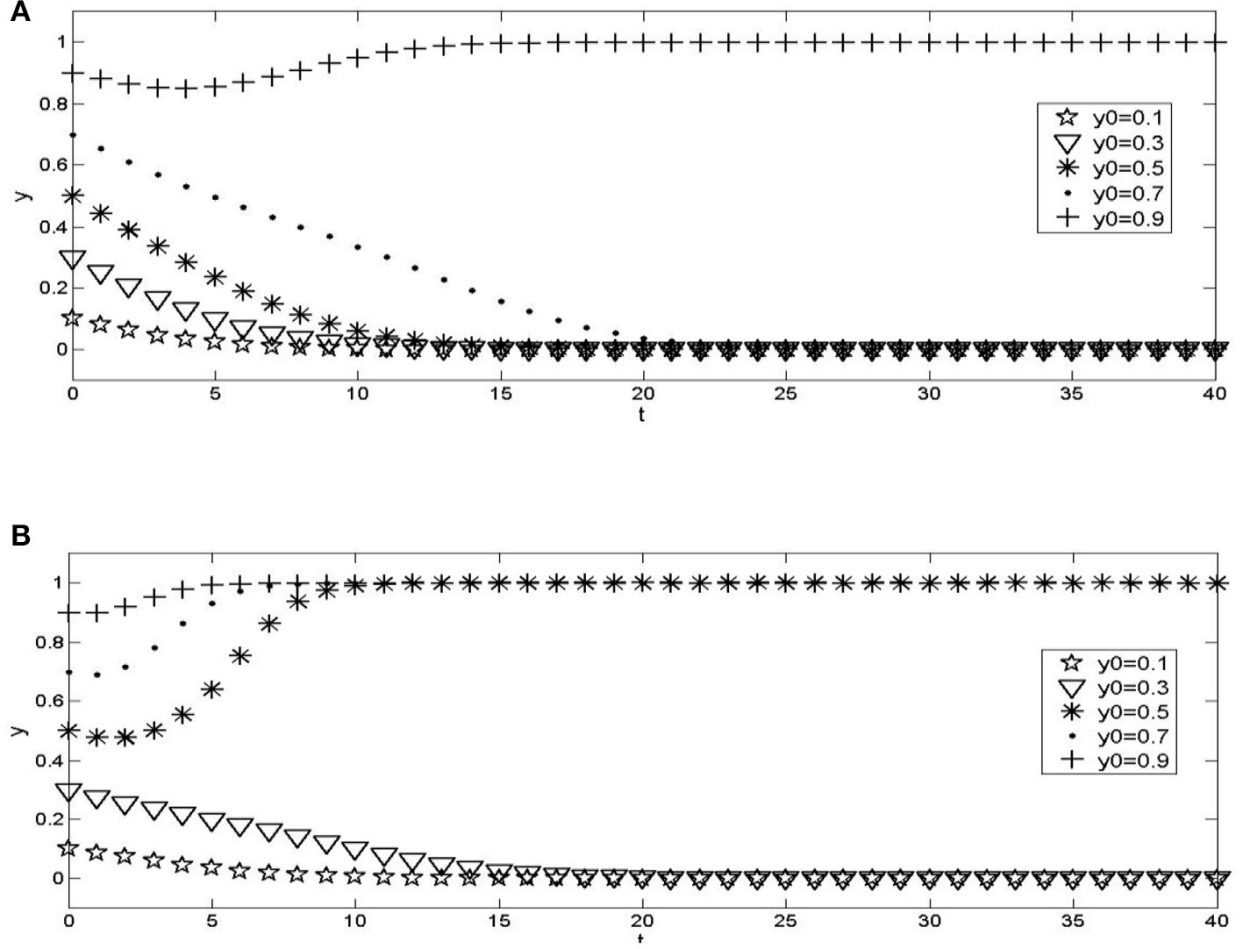
The impact of changes in government incentives given by urban hospitals and community health service institutions on the evolution results. **(A)** *G*_3_ = 0, *G*_4_ = 0; **(B)** *G*_3_ = 0.5, *G*_4_ = 0.6.

1. The influence of the proportion change of the initial population on the evolution results. Assuming parameters, *C*_3_ = 2, *C*_4_ = 1*C*_5_ = 1.5, *C*_6_ = 0.4, *E*_3_ = 2.5, *E*_4_ = 1.3, *E*_5_ = 2.2, *E*_6_ = 0.9, *E*_7_ = 0.21, *G*_1_ = 0.6, *G*_2_ = 0.4, *G*_3_ = 0.1, *G*_4_ = 0.15, *a* = 0.5, the numerical experimental results are shown in Figure 2. It can be seen that the communication process of the behavior strategies of the two types of institutions has path dependence. The convergence curves under different initial ratios will not overlap before reaching equilibrium. The time to converge to the equilibrium state is affected by the initial strategies of urban hospitals and community health service institutions. The closer the proportion of the selection strategy is to the equilibrium point, the faster the convergence speed of the system is.
2. The government monitors the impact of changes in probability on the evolution results. The values of a parameter *a* are 0.2 and 0.8, respectively. The importance of other parameters are the same as those in Figure 2A. The numerical experimental results are shown in Figure 3. Compared with Figure 2A, it can be seen that the change of monitoring intensity has a significant impact on the evolution results of the system. When the probability of government monitoring becomes smaller (Figure 3A), the possibility of the system converging to the wrong mode becomes more significant, and the probability of cooperation between urban and rural medical and health institutions decreases; On the contrary, when the government increases the monitoring probability (Figure 3B), it can be found that the system converges to a benign state, indicating that when the government's monitoring probability is large enough, the cooperation system between urban hospitals and community health service institutions can evolve to an ideal state.
3. The impact of government incentives on the evolution results after the cooperation between urban hospitals and community health service institutions. The values of parameters *G*_3_ and *G*_4_ are 0, that is, the amount of reward given by the government after the urban and rural medical and health institutions adopt the cooperation strategy is 0; The values of parameters *G*_3_ and *G*_4_ are 0.5 and 0.6, respectively, that is, the government further increases the reward after the cooperation of urban and rural medical and health institutions. The values of other parameters are the same as those in Figure 2A. The numerical experimental results are shown in Figure 4. Compared with Figure 2A, when the government does not give rewards after the cooperation between urban and rural medical and health institutions (Figure 4A), the possibility of the system converging to a lousy state increases; When the reward given by the government increases after the cooperation between urban and rural medical and health institutions (Figure 4B), the system gradually tends to the ideal model, and the convergence speed gradually increases.

## Discussions

From the results we can concluded that the cooperative behavior of both parties is affected by the willingness of urban hospitals and community health service institutions, the willingness of one party will affect the strategy choice behavior of the other party. It also can also be inferred that most patients choose to avoid the “first diagnosis at the grass-roots level” and go directly to large hospitals due to insufficient grass-roots medical service capacity and limited resources, or the unlimited expansion of the scale of urban hospitals and the reluctance of urban hospital doctors to practice in community health service institutions make it impossible for community health service institutions and urban hospitals to realize an effective cooperation mechanism, This has further hindered the cooperation between urban and rural medical and health institutions. We can also concluded that it is necessary to strictly control and monitor the means of vicious competition between medical and health institutions to promote urban and rural medical and health institutions to adopt cooperative strategies. The results also shows that the government's reward mechanism will significantly impact the cooperation strategy of urban and rural medical and health institutions, and the greater the degree of reward, the more pronounced the effect of inter-agency cooperation.

Hence the policymakers are implored to consider the following regulatory measures to strengthen the collaboration between urban and rural medical and health institutions. Firstly, the need to highlight the government's overall responsibility. This can be done by supporting the government's transfer payments to various medical and health institutions while prioritizing the capacity building of community health service institutions to provide medical services. They can also increase public awareness and direct patients toward standardized medical treatment and gradually achieve hierarchical diagnosis and treatment. Secondly, the need to strengthen government supervision is recommended. Decision-makers are encouraged to thwart medical and health institutions from engaging in a vicious competition to maximize profits or improve their medical ability. They can again impose strict measures and increase the amount of compensation for institutions that use cooperation strategies. Finally, enhancing cooperation mechanisms is also recommended. The strategic behavior of urban hospitals and community health service institutions impacts the cooperation mechanism of urban and rural medical and health institutions. As a result, it is necessary to construct a cooperation mechanism between them, alleviate interest contradictions, improve relevant rules and regulations, realize benign interaction between them, participate in cooperative activities together, and ultimately achieve a win-win situation.

## Conclusions

With the growing issue of insufficient cooperation between urban and rural medical and health institutions, this article analyzes the competition and cooperation relationships between urban hospitals and rural health service institutions. The study further discusses the system's evolution and stability strategy using the evolutionary game method and conducts numerical simulations. The findings show that establishing a cooperation mechanism between urban and rural medical and health institutions depends on the main body's initial strategy. It is also associated with the likelihood of government supervision and the number of penalties for violations, including the cost of infringements and incentives provided by the government after cooperation.

## Limitations

The parameter value in the numerical experiment was estimated, which was assumed according to the actual situation in the division of labor and cooperation of medical institutions in a region. It was representative, but it cannot be fully applicable to all regions. In the future, the research scope will be further expanded and the parameter value will be updated to make the research more referential.

## Author's Note

XX: Holds a PhD in Management Science with research and teaching interest in Health Economics and Health Policy Management. He has 10 years of teaching and research experience in this area. He is currently a professor at the School of Management at Jiangsu University.

JL: He is a graduate student of Jiangsu University. He has 6 years of learning experience and 2 years of research experience in the area of health reform. He has published two academic papers on hospital management and medical reform currently.

SA-W: Holds a PhD in Management Science with 8 years of teaching and research experience in Human Resource in Healthcare, Social Policy, and Healthcare Management. She is currently a Post-Doctoral of the Institute of Systems Engineering in Jiangsu University and Senior Foreign Expert at the Centre for Health and Public Policy Research of Jiangsu University.

HA: Holds a PhD in Management Science with a research interest in International Management, Public Management, and Health and Development Policy. He is currently a postdoctoral fellow of the Institute of Systems Engineering and a Senior Foreign Expert at the Centre for Health and Public Policy Research of Jiangsu University and the Shanghai Normal University. He has 10 years teaching and research experience in Management Science, Public Management and Development Policy.

LZ: Professor of Public Management, Head of Medical Insurance and Public Policy Research Center in Jiangsu Province (PRC). He has 30 years of teaching and research experience and published articles in Social Policy, Public Management and Healthcare Policy Management.

## Data Availability Statement

The raw data supporting the conclusions of this article will be made available by the authors, without undue reservation.

## Author Contributions

XX and JL are responsible for the conception and design of the study. XX collected and performed the analysis. LZ was consulted during the analysis and interpretation process. XX, JL, and SA-W led the drafting of the manuscript. SA-W, HA, and LZ were involved in critically revising the manuscript for important intellectual content. All authors carried out the interpretation of data, read, and approved the final manuscript.

## Funding

The study was funded by the Natural Science Foundation of China (7194066) and the Social Science Fund Project of Jiangsu Province (20SHD002).

## Conflict of Interest

The authors declare that the research was conducted in the absence of any commercial or financial relationships that could be construed as a potential conflict of interest.

## Publisher's Note

All claims expressed in this article are solely those of the authors and do not necessarily represent those of their affiliated organizations, or those of the publisher, the editors and the reviewers. Any product that may be evaluated in this article, or claim that may be made by its manufacturer, is not guaranteed or endorsed by the publisher.
